# Measuring information alignment in hyperscanning research with representational analyses: moving beyond interbrain synchrony

**DOI:** 10.3389/fnhum.2024.1385624

**Published:** 2024-07-25

**Authors:** Manuel Varlet, Tijl Grootswagers

**Affiliations:** ^1^The MARCS Institute for Brain, Behaviour and Development, Western Sydney University, Sydney, NSW, Australia; ^2^School of Psychology, Western Sydney University, Sydney, NSW, Australia; ^3^School of Computer, Data and Mathematical Sciences, Western Sydney University, Sydney, NSW, Australia

**Keywords:** hyperscanning, EEG, interbrain synchrony, social interaction, representational alignment, information content

## Abstract

Hyperscanning, which enables the recording of brain activity from multiple individuals simultaneously, has been increasingly used to investigate the neuropsychological processes underpinning social interaction. Previous hyperscanning research has primarily focused on interbrain synchrony, demonstrating an enhanced alignment of brain waves across individuals during social interaction. However, using EEG hyperscanning simulations, we here show that interbrain synchrony has low sensitivity to information alignment across people. Surprisingly, interbrain synchrony remains largely unchanged despite manipulating whether two individuals are seeing same or different things at the same time. Furthermore, we show that hyperscanning recordings do contain indices of interpersonal information alignment and that they can be captured using representational analyses. These findings highlight major limitations of current hyperscanning research and offer a promising alternative for investigating interactive minds.

## 1 Introduction

Understanding how social interactions dynamically shape human mind and behavior and vice versa is a key question in cognitive neuroscience and psychology (Sebanz et al., 2006; Hari et al., 2015). Hyperscanning, which enables the recording of the brain activity of multiple individuals at the same time, has been increasingly used to address this question. Hyperscanning research revealed enhanced interbrain synchrony between individuals interacting socially, which has been argued to reflect resonant minds facilitating cooperation and communication (Dumas et al., 2010; Dikker et al., 2017; Pérez et al., 2017; Goldstein et al., 2018; Reindl et al., 2018). However, here we highlight an important limitation of interbrain synchrony measures by showing that these measures indexing the alignment of neural activity patterns across individuals have low sensitivity to their Information content. We show that representational analyses, the framework of Representational Similarity Analysis (RSA) in particular, is a promising alternative to address this issue and better index information alignment across individuals in hyperscanning research.

## 2 Interbrain synchrony does not measure information alignment

Interbrain synchrony has been investigated in hyperscanning research using a range of neuroimaging techniques including Electroencephalography (EEG), Magnetoencephalography (MEG), functional Magnetic Resonance Imaging (fMRI), and functional Near-InfraRed Spectroscopy (fNIRS). Numerous measures have been used to index interbrain synchrony in hyperscanning studies generally based on amplitude correlation and phase locking (Czeszumski et al., 2020). For EEG and MEG, enabling access to neural processing at faster time scales, these analyses have also focused on the amplitude and phase of neural oscillations, with larger emphases on activity in the alpha (8–12 Hz) and beta (13–30 Hz) bands (Dumas et al., 2010; Goldstein et al., 2018). See Czeszumski et al. (2020) for a comprehensive review of hyperscanning and interbrain synchrony measures.

While all these methods are relevant to measure synchrony in general, the processes captured by interbrain synchrony in the context of hyperscanning research remain unclear. The causal nature of this phenomenon has been the main source of concerns so far, whether interbrain synchrony directly drives synchronised mind and behavior or interbrain synchrony is simply caused by synchronised mind and behavior (Burgess, 2013; Hari et al., 2013; Hamilton, 2021; Novembre and Iannetti, 2021; Holroyd, 2022). If synchronised mind and behavior causes interbrain synchrony, then this suggests that there is no direct brain-to-brain coupling between individuals, and interbrain synchrony is the result of similar sensory and cognitive processes driven by shared environment and task. As pointed out by Hamilton (2021), a lack of direct brain-to-brain coupling may not be a critical issue because interbrain synchrony measures are still of interest for hyperscanning research if they provide insight into the alignment of sensory information and cognitive processes across individuals while socially interacting.

**Figure 1:**
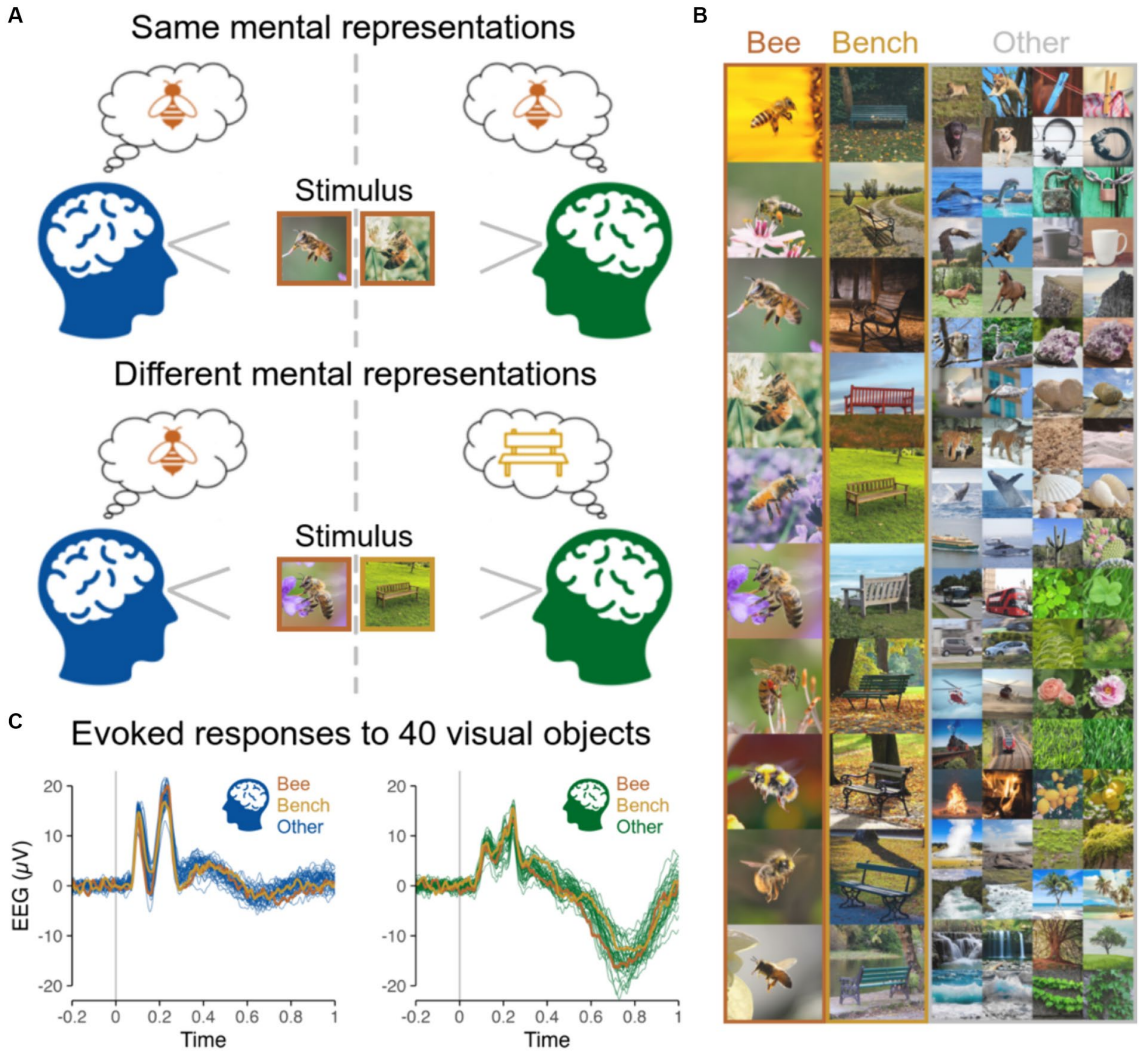
**(A)** Same vs. different mental representations across two subjects induced by the presentation of same vs. different objects at the same time. **(B)** Stimuli presented to subjects individually in Shatek et al. (2022), including 40 different categories of objects, each with 10 different images presented 10 times, while EEG used here to run hyperscanning simulations was recorded. The 10 images are shown for the categories Bee and Bench, and 2 out of the 10 images for each of the remaining 38 categories are shown in the ‘Other’ column. **(C)** EEG evoked responses for two representative subjects (at channel POz) for the 40 categories of objects averaged across all images and repetitions highlighting the low magnitude of variations related to image content.

**Figure 2:**
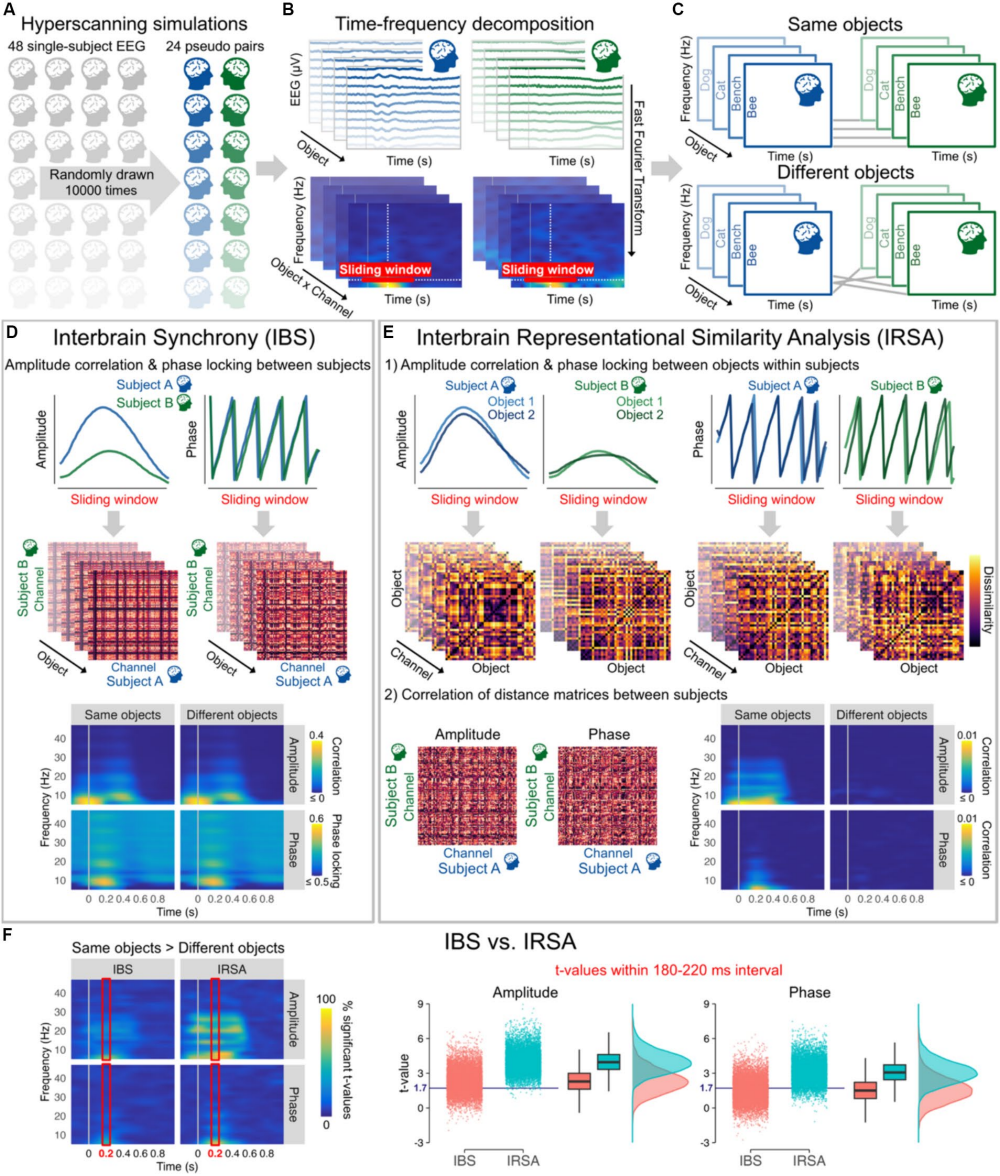
**(A)** 10,000 hyperscanning simulations based on individual 128-channel EEG recordings from Shatek et al. (2022), each simulation including 24 pseudo pairs randomly drawn from the 48 subjects available. **(B)** EEG data were average referenced, bandpass filtered between 0.1 and 100 Hz, and downsampled to 250 Hz. Amplitude and phase were then estimated for each channel and all 40 objects (averaged across all stimuli and repetitions within categories) from 6 to 46 Hz every 10 ms using a 500 ms sliding Hanning window yielding a 2 Hz frequency resolution (Oostenveld et al., 2011). **(C)** Objects presented to the two subjects of each pair were either kept the same (Same objects) or made different by shuffling their order (Different objects). **(D)** Interbrain synchrony (IBS) was computed at each 10 ms time step and frequency bin as the amplitude (Pearson) correlation and phase locking (relative phase mean vector length) between the two subjects of the pairs for all combinations of channels within a 510 ms sliding window from 0.2 to 1 s before/after stimulus onset. The resulting 128 × 128 channel interbrain connectivity matrices for each object were then averaged to obtain a single amplitude correlation and phase locking value at each time step and frequency bin for Same objects and Different objects. Interbrain connectivity matrices are from a representative pseudo pair (at 10 Hz and 200 ms after stimulus onset) and time-frequency maps correspond to the grand average of all pseudo pairs and simulations. **(E)** For Interbrain Representational Similarity Analysis (IRSA), amplitude correlation and phase locking were first calculated between all pairs of objects within a 510 ms sliding window from 0.2 to 1 s before/after stimulus onset to obtain dissimilarity matrices. Amplitude dissimilarity was calculated as 1 – Pearson correlation and phase dissimilarity was calculated as 1 – phase locking (relative phase mean vector length). We then computed the (Spearman) correlation between dissimilarity matrices across the two subjects for all combinations of channels. The resulting 128 × 128 channel interbrain connectivity matrices were averaged to obtain a single correlation value at each time step and frequency bin for Same objects and Different objects. The figure shows example dissimilarity matrices and interbrain connectivity matrices from a representative pseudo pair (at 10 Hz and 200 ms after stimulus onset, channel POz for the dissimilarity matrices). Time-frequency maps correspond to the grand average of all pseudo pairs and simulations. **(F)** Time-frequency maps (averaged across all possible combinations of channels) represent the percentage of significant t-values from the 10,000 simulations for Same objects vs. Different objects (*t*-values >1.714, critical *t*-value for one-tailed *t*-tests with 24 observations, alpha = 0.05) for amplitude and phase data as a function of IBS and IRSA, with brighter colours indicating that large proportions of simulations yielded significant differences. The right panels represent the mean *t*-values within 180–220 ms (time interval showing highest percentages of significant *t*-values in line with peak decoding accuracy in object literature) from the 10,000 simulations for Same objects vs. Different objects after averaging correlation and phase locking data across all frequency bins. The blue horizontal line indicates the critical *t*-value. These plots show that the same vs. different objects differences were observed only in around 50% of simulations using IBS, but in more than 80% of simulations when using IRSA.

However, here we present results showing that interbrain synchrony measures do not effectively index information alignment between individuals because these measures are largely content agnostic. We leveraged a publicly available EEG dataset from the visual object recognition literature with 48 participants who were presented with 400 images from 40 categories (Shatek et al., 2022). All recordings were made individually with a 128-channel BrainVision actichamp EEG system. We used this dataset to run hyperscanning simulations to assess the sensitivity of interbrain synchrony to information alignment by comparing interbrain synchrony when two individuals see the same things (Same objects) vs. different things (Different objects) at the same time (Figures 1, 2).

We ran 10,000 hyperscanning simulations with 24 pseudo pairs randomly drawn from the 48 subjects available (Figure 2). Interbrain synchrony was computed for a wide range of frequencies as amplitude correlation and phase locking across subjects for all possible combinations of channels (Czeszumski et al., 2020). Results were averaged across all possible combinations of channels to capture any potential changes in interbrain synchrony. With optimal control of the timing and content of the presented stimuli, the results from the simulations revealed that interbrain synchrony has low sensitivity to information alignment across individuals. Amplitude correlation and phase locking values remained largely unchanged whether people were seeing the same object or not (Figure 2D). *t*-values after stimulus presentation across all frequencies obtained from the 10,000 simulations show that difference between Same objects and Different objects would in most cases be statistically undetectable despite a sample size at least similar (i.e., 24 pairs) to those generally used in the literature (Figure 2F). Figure 2F depicting *t*-values on averaged amplitude and phase values across frequencies within 180 and 220 ms after image presentation – time window yielding highest percentages of significant *t*-values and peak decoding accuracy in object literature in general (Grootswagers et al., 2019; Shatek et al., 2022) – shows that barely 50% of the simulations reached statistical significance for interbrain synchrony (IBS). See legend of Figure 2 and publicly available code at https://osf.io/etx64/ for further methodological details.

## 3 Interbrain representational similarity analysis to effectively measure information alignment

The Representational Similarity Analysis (RSA) framework has received significant interest in the field of cognitive neuroscience (Kriegeskorte et al., 2008; Haxby et al., 2014), including more recently in social neuroscience and the intersubject correlation community (Nastase et al., 2019; Popal et al., 2019), but has not been used yet in hyperscanning research. This analysis is based on the computation of Representational (Dis)similarity Matrices (RDMs) to abstract from the patterns of neural activity themselves and characterise their informational content, allowing testing how two different systems quantitatively relate to each other by comparing their RDMs. RSA makes it possible to compare the responses from different systems, including responses recorded with different neuroimaging systems, neuroimaging and behavioral responses, as well as responses from different individuals, as shown here.

We computed Interbrain RSA (IRSA) using the same amplitude and phase data as IBS (Figure 2E). RDMs that encode information content and abstract from activity patterns were first computed separately for each subject and channel by calculating the amplitude correlation and phase locking for all pairs of stimuli. This approach based on (dis)similar temporal patterns in time-frequency data separately for each channel differs from other approaches often used with RSA (Kriegeskorte et al., 2008; e.g., Grootswagers et al., 2017; Shatek et al., 2022), but allows here to directly compare between IRSA and IBS. The results of the simulations show that information alignment can be captured in EEG hyperscanning with better sensitivity using IRSA. IRSA values decrease dramatically when shuffling the stimuli (Figure 2E), as reflected in differences between Same object vs. Different objects being statistically detected more consistently than IBS with statistical significance being reached in most simulations (Figure 2F). See legend of Figure 2 and publicly available code at https://osf.io/etx64/ for further methodological details.

## 4 IBS vs. IRSA: statistical comparison

Statistical analyses on the 10,000 simulations indicated that *t*-values testing differences between Same vs. Different objects in the 180–220 ms selected time window, as depicted in Figure 2F, were significantly higher for IRSA than IBS for both amplitude, *t*(19998) = 117.22, *p* < 0.0001, *d* = 1.66, BF_10_ > 1,000, and phase, *t*(19998) = 109.31, *p* < 0.0001, *d* = 1.55, BF_10_ > 1,000, showing that IRSA has higher sensitivity to content shuffling than IBS. These effects held beyond the selected time window as indicated by significantly higher *t*-values for IRSA than IBS for both amplitude, *t*(19998) = 139.21, *p* < 0.0001, *d* = 1.97, BF_10_ > 1,000, and phase, *t*(19998) = 95.87, *p* < 0.0001, *d* = 1.36, BF_10_ > 1,000, when conducting these analyses on a larger time interval from 0 to 500 ms after stimulus presentation. Large effect sizes observed here demonstrate a critical advantage of IRSA over IBS to index information alignment from amplitude and phase data in hyperscanning research.

## 5 Perspectives and challenges

Using hyperscanning simulations with well-controlled visual stimuli, our work shows that content-related information in hyperscanning research is not effectively captured by interbrain synchrony measures. This contrasts with previous research that found reliable decrease in interbrain synchrony when shuffling data across trials and/or pairs (Zamm et al., 2021, 2024; Reindl et al., 2022), which our results suggest might most likely be due to breaking the alignment of the timing of sensory and cognitive processes occurring in shared tasks and environments rather than the alignment of their information content. Shuffling information content while keeping timing constant in our simulations only marginally decreased interbrain synchrony whereas changes in timing resulting in large variations in neural signals would have a strong influence (Burgess, 2013; Holroyd, 2022). Examining and controlling for timing (dis)alignment of sensory, cognitive, and motor responses when shuffling across pairs and/or trials will be essential in future research to better understand the processes reflected by interbrain synchrony.

More generally, these results support the hypothesis that interbrain synchrony is caused by synchronised mind and behavior rather than the opposite, interbrain synchrony causing synchronised mind and behavior. Showing that interbrain synchrony does not uniquely capture sensory and cognitive processes supporting social interactions, our results suggest that interbrain synchrony cannot be a causal mechanism (underpinned by direct brain-to-brain coupling), and importantly, might be at best a poor proxy of synchronised sensory and cognitive processes supporting social interactions (Hamilton, 2021; Novembre and Iannetti, 2021). To be an effective top-down mechanism enabling the myriads of social behaviors observed every day, interbrain synchrony would not only require reflecting reliable timing information but also content information, which is not supported by our hyperscanning simulations.

The lack of unique and direct relationship between interbrain synchrony and synchronised sensory and cognitive processes during social interactions might explain part of the discrepancies in hyperscanning literature, including interbrain synchrony not being observed in some studies despite participants interacting and synchronising with each other (Liu et al., 2018; Czeszumski et al., 2020; Newman et al., 2024). While further research with a wider range of sensory and cognitive processes and neuroimaging techniques will be needed to expand our work beyond EEG visual evoked responses, the limitations of interbrain synchrony revealed here add to the growing concerns having been expressed in the field (Burgess, 2013; Hari et al., 2013; Hamilton, 2021; Novembre and Iannetti, 2021; Holroyd, 2022).

Our results highlight representational analyses as a powerful alternative to synchrony measures to better index information alignment between individuals. Enabling information content to be compared across individuals, even while obtained from different (neuroimaging) systems, these analyses will have critical advantages for future hyperscanning research. While implementing representational analyses is relatively straightforward when having time-locked trials with many different stimuli, moving into the representational space in more naturistic tasks as often used in hyperscanning research will be more challenging. Advancing representational analyses methods for non-time locked data will be needed to reach full capacity of hyperscanning and enable a step change in understanding the sensory and cognitive processes supporting real-time social interactions.

## Data availability statement

The datasets presented in this study can be found in online repositories. The names of the repository/repositories and accession number(s) can be found at: https://osf.io/etx64/.

## Ethics statement

The studies involving humans were approved by University of Sydney Human Research Ethics Committee. The studies were conducted in accordance with the local legislation and institutional requirements. The participants provided their written informed consent to participate in this study.

## Author contributions

MV: Conceptualization, Methodology, Writing – original draft. TG: Conceptualization, Methodology, Writing – review & editing.
